# Supplementary material for: The Systems Biology Research Tool: evolvable open-source software
Source: BMC Syst Biol. 2008 Jun 29;2:55. doi: 10.1186/1752-0509-2-55 (PMC2446383; doi:10.1186/1752-0509-2-55)
Supplement: Additional file 1 — SBRT Archive. An archive of the current version of the Systems Biology Research Tool. [file 1752-0509-2-55-S1.zip › sbrt-1.4.0/doc/developers_guide/api/sbrt/kernel/fba/FluxConstraints.html]

FluxConstraints


|  |  |  |
| --- | --- | --- |
| **Overview** \| **Package** \| **Class** \| **Use** \| **Tree** \| **Deprecated** \| **Index** \| **Help** | |  |
| **PREV CLASS**   **NEXT CLASS** | **FRAMES**    **NO FRAMES**     **All Classes** |  |
| SUMMARY: NESTED \| FIELD \| CONSTR \| METHOD | DETAIL: FIELD \| CONSTR \| METHOD |  |


---


## sbrt.kernel.fba Class FluxConstraints

```
java.lang.Object
  sbrt.kernel.fba.FluxConstraints
```

---

``` public class FluxConstraints extends java.lang.Object ```

This class is used to group a set of flux constraints used in
Flux Balance Analysis.

Typically, a constraint in FBA only applies to the flux of a
single chemical reaction. This type of constraint is referred
to here as a "flux constraint".

It is also possible, though, to constrain the value of a
mathematical expression containing one or more fluxes. This
type of constraint is referred to here as an "expression constraint", or
sometimes as a "reaction name expression constraint".

**Author:**
:   This class was written by Jeremiah Wright while in the Wagner lab.

---

**Constructor Summary**

`FluxConstraints(java.util.Map<java.lang.String,Interval> fluxConstraints)`
  
          Constructs a set of FBA constraints from the provided argument.

`FluxConstraints(java.util.Map<java.lang.String,Interval> fluxConstraints,
java.util.Map<? extends MathExpr<java.lang.String>,Interval> exprConstraints)`
  
          Constructs a set of FBA constraints from the provided arguments.


| **Method Summary** | |
| --- | --- |
| `Interval` | `getConstraint(MathExpr<java.lang.String> rxnExpr)`             Returns the constraint of the provided mathematical expression of reaction names. |
| `Interval` | `getConstraint(java.lang.String rxnName)`             Returns the flux constraint for the provided reaction. |
| `UnmodifiableMap<MathExpr<java.lang.String>,Interval>` | `getExprConstraints()`             Returns the constraints for mathematical expressions of fluxes. |
| `UnmodifiableMap<java.lang.String,Interval>` | `getFluxConstraints()`             Returns the flux constraints for single reactions. |
| `int` | `total()`             Returns the total number of elements contained in this set of constraints. |

**Methods inherited from class java.lang.Object**

`clone, equals, finalize, getClass, hashCode, notify, notifyAll, toString, wait, wait, wait`

**Constructor Detail**

### FluxConstraints

```
public FluxConstraints(java.util.Map<java.lang.String,Interval> fluxConstraints,
                       java.util.Map<? extends MathExpr<java.lang.String>,Interval> exprConstraints)
```

:   Constructs a set of FBA constraints from the provided arguments.

    **Parameters:**: `fluxConstraints` - reactions names mapped to their corresponding flux constraint.: `exprConstraints` - mathematical expressions of reaction names mapped to their corresponding constraint.

---


### FluxConstraints

```
public FluxConstraints(java.util.Map<java.lang.String,Interval> fluxConstraints)
```

:   Constructs a set of FBA constraints from the provided argument.

    **Parameters:**: `fluxConstraints` - reactions names mapped to their corresponding flux constraint.


**Method Detail**

### getFluxConstraints

```
public UnmodifiableMap<java.lang.String,Interval> getFluxConstraints()
```

:   Returns the flux constraints for single reactions.

    :   **Returns:**: the flux constraints for single reactions.

---


### getExprConstraints

```
public UnmodifiableMap<MathExpr<java.lang.String>,Interval> getExprConstraints()
```

:   Returns the constraints for mathematical expressions of fluxes.

    :   **Returns:**: the constraints for mathematical expressions of fluxes.

---


### getConstraint

```
public Interval getConstraint(java.lang.String rxnName)
```

:   Returns the flux constraint for the provided reaction.

    :   **Parameters:**: `rxnName` - the name of the reaction whose flux constraint is to be returned. **Returns:**: the flux constraint for the provided reaction, or `null` if the reaction is not present in this set.

---


### getConstraint

```
public Interval getConstraint(MathExpr<java.lang.String> rxnExpr)
```

:   Returns the constraint of the provided mathematical expression of
    reaction names.

    :   **Parameters:**: `rxnExpr` - the mathematical expression of reaction names whose constraint is to be returned. **Returns:**: the constraint of the provided mathematical expression of reaction names, or `null` if the expression is not present in this set.

---


### total

```
public int total()
```

:   Returns the total number of elements contained in this set of constraints.

    :   **Returns:**: the total number of elements contained in this set of constraints.


---


|  |  |  |
| --- | --- | --- |
| **Overview** \| **Package** \| **Class** \| **Use** \| **Tree** \| **Deprecated** \| **Index** \| **Help** | |  |
| **PREV CLASS**   **NEXT CLASS** | **FRAMES**    **NO FRAMES**     **All Classes** |  |
| SUMMARY: NESTED \| FIELD \| CONSTR \| METHOD | DETAIL: FIELD \| CONSTR \| METHOD |  |


---
